# Dual immune modulation of microglia in viral encephalitis: current understanding and future perspectives

**DOI:** 10.3389/fmolb.2025.1695058

**Published:** 2025-10-27

**Authors:** Li Zong, Pei Chen, Jing Shi, Huijie Chen, Wenwen Lin, Guanyong Ou, Xuxiang Chen

**Affiliations:** ^1^ Department of Rehabilitation Medicine, The People’s Hospital of Xishuangbanna Dai Nationality Autonomous Prefecture, Xishuangbanna, Yunnan, China; ^2^ Department of Infectious Disease, The Eighth Affiliated Hospital of Sun Yat-sen University, Shenzhen, Guangdong, China; ^3^ Naval Medical Center, Naval Medical University, Shanghai, China; ^4^ National Key Laboratory of Immunity and Inflammation, Shanghai, China; ^5^ Translational Medical Research Center, Naval Medical University, Shanghai, China; ^6^ Department of Neurology, The People’s Hospital of Kaifeng, Kaifeng, Henan, China; ^7^ Department of Pathology, Shenzhen Hospital of Southern Medical University, Shenzhen, China; ^8^ Shenzhen Key Laboratory of Pathogen and Immunity, Shenzhen Third People’s Hospital, Second Affiliated Hospital, School of Medicine, Southern University of Science and Technology, Shenzhen, China; ^9^ Department of Emergency, The Eighth Affiliated Hospital of Sun Yat-sen University, Shenzhen, Guangdong, China

**Keywords:** viral encephalitis, central nervous system, microglia, neuroinflammation, activation, dual function

## Abstract

Viral encephalitis, characterized by inflammation of the brain parenchyma, poses a significant threat to public health due to its high rates of morbidity and mortality. Microglia, the central nervous system’s resident immune cells, are crucial in the pathophysiology and development of this condition. These microglia exhibit a dual function, being involved in both neuroprotection and neurotoxicity during viral encephalitis. To address this complex interplay, targeted therapeutic strategies that modulate microglia activation state have emerged as a promising approach. These strategies aim to either inhibit excessive microglia activation or promote their neuroprotective functions. By targeting microglia, these therapies hold the potential to improve outcomes for patients with viral encephalitis. This review synthesizes current evidence revealing that microglial responses during viral encephalitis exhibit context-dependent heterogeneity that extends beyond traditional M1/M2 paradigms. Critically, our review reveals a significant translational gap, with no current clinical trials investigating microglial-targeted therapies for viral encephalitis despite promising preclinical evidence. This review provides a comprehensive framework for understanding microglial complexity in viral encephalitis and establishes research priorities for advancing these insights toward clinical application.

## 1 Introduction

Viral encephalitis is an inflammatory condition in the central nervous system (CNS) that primarily affects the brain parenchyma (Venkatesan and Murphy, 2018; Venkatesan et al., 2013). It can be caused by a wide range of RNA and DNA viruses, including Japanese encephalitis virus (JEV), West Nile virus (WNV), herpes simplex virus (HSV) and enteroviruses (Venkatesan and Murphy, 2018; Costa and Sato, 2020; McMillan et al., 2023). The infection may manifest as a primary viral infection, a reactivation of latent viral infection, or as a result of viral dissemination (Granerod et al., 2010).

The global incidence of viral encephalitis varies depending on the virus and geographical location, with approximately 3.5–7.4 cases per 100,000 individuals annually (Venkatesan et al., 2013; Granerod et al., 2010; Vora et al., 2014). It poses significant public health challenges, particularly in developing countries, due to high morbidity and mortality rates (Solomon et al., 2012). Clinical manifestations of viral encephalitis can range from mild, nonspecific symptoms such as fever, headache, and lethargy to severe neurological complications, including seizures, cognitive difficulties, and altered consciousness (Venkatesan and Murphy, 2018; Whitley and Gnann, 2002). Despite the availability of antiviral therapies for some viral encephalitis etiologies, the prognosis remains poor, particularly for HSV encephalitis, which has a mortality rate of approximately 70% without treatment (Solomon et al., 2012).

Microglia, the resident immune cells of the CNS, are crucial not only for maintaining neuronal homeostasis but also in the context of infections, such as viral encephalitis (Ginhoux and Prinz, 2015; Nayak et al., 2014). Microglial activation and subsequent immune responses can both protect the host by limiting viral replication and spread or exacerbate the infection by causing neuroinflammation and neuronal damage (Nayak et al., 2014; Salter and Stevens, 2017). Therefore, understanding the role and functions of microglia during viral encephalitis is critical for the development of targeted therapeutics to modulate their neuroprotective functions while minimizing neurotoxic effects (Mariani and Kielian, 2009).

This review aims to provide a comprehensive overview of the current knowledge on microglial involvement in viral encephalitis, focusing on their roles in pathogen recognition, orchestrating antiviral immune responses, and their contribution to neuroinflammation and blood-brain barrier disruption. Additionally, we will discuss the dual role of microglia in viral encephalitis, delineating their neuroprotective and neurotoxic functions. Finally, prospective microglia-targeting treatment approaches and potential future research objectives in this area will be discussed.

## 2 Overview of microglia

### 2.1 Origin and development

Microglia belong to the mononuclear phagocyte system, a group of myeloid cells that includes monocytes, macrophages, and dendritic cells (Borst et al., 2021; Prinz and Priller, 2014). Unlike other cell types in the CNS, microglia are relatively long-lived, maintaining their population through self-renewal rather than relying on the turnover of circulating precursors (Prinz and Priller, 2014). Microglia play a crucial role in immune surveillance, sustaining tissue homeostasis, and responding to injury or virus infection in the CNS (Prinz and Priller, 2014; Erny et al., 2015). They are highly dynamic cells that constantly survey their microenvironment and can rapidly respond to changes by altering their morphology and function (Nimmerjahn et al., 2005).

The origin of microglia has been a subject of debate, and Hortega’s classical view suggests that microglia originate from the mesoderm and enter the brain during late embryonic development when blood vessels form (Boullerne and Feinstein, 2020). During early embryonic development, microglia arise from primitive macrophages in the yolk sac and migrate to the developing brain (Ginhoux et al., 2010). Once they reach the CNS, they differentiate into ramified cells with highly branched processes that continuously survey their surroundings (Nimmerjahn et al., 2005). Microglia are characterized by their unique molecular signature, which includes various cell surface markers such as CD11b, CD45, and Iba1 (Ginhoux et al., 2010; Lan et al., 2017).

In addition to the role in immune surveillance, microglia also actively participate in shaping the developing brain. They contribute to neural circuit refinement, synaptic pruning, and the elimination of excess synapses during development (Paolicelli et al., 2011). Microglia also play a role in neurogenesis and neuroinflammation, and their malfunction is implicated in various neurological disorders, such as Alzheimer’s disease (AD) and multiple sclerosis (MS) (Prinz and Priller, 2014; Hickman et al., 2018).

### 2.2 Functions in the CNS

Microglia play multiple roles in the CNS, including maintenance of homeostasis, surveying the microenvironment, synaptic pruning, and mediating adaptive and innate immune responses (Nayak et al., 2014; Salter and Stevens, 2017). Under physiological conditions, microglia exhibit a ramified morphology characterized by a small cell body with numerous branched processes, allowing them to constantly monitor their surroundings for any potential threats (Nimmerjahn et al., 2005). In response to injury, infection, or other disruptions of CNS homeostasis, microglia undergo morphological and functional changes, becoming activated, phagocytic, and releasing cytokines and chemokines (Wolf et al., 2017; Garaschuk and Verkhratsky, 2019; Kettenmann et al., 2011).

Activated microglia can phagocytose cellular debris, pathogens, and damaged neurons, contributing to tissue repair and clearance of harmful substances (Salter and Stevens, 2017). They also release pro-inflammatory factors, such as interleukin-1 beta (IL-1β) and tumor necrosis factor-alpha (TNF-α), which recruit immune cells to the site of injury and initiate an inflammatory response (Kettenmann et al., 2011). In addition to their immune functions, microglia actively participate in synaptic pruning and refinement of neural circuits during development (Paolicelli et al., 2011). They remove excess synapses, allowing for the proper wiring of neuronal connections and optimizing neural circuitry (Schafer et al., 2012). Dysfunction or dysregulation of microglial pruning processes has been implicated in neurodevelopmental disorders, such as autism spectrum disorders and schizophrenia (Schafer et al., 2012). Furthermore, microglia are involved in neurogenesis and neuroplasticity, influencing the formation and maintenance of new neurons and synaptic connections (Sierra et al., 2010). They release growth factors and neurotrophic factors that support neuronal survival, differentiation, and synaptic plasticity (Sierra et al., 2010).

However, excessive or prolonged activation of microglia can lead to more severe neuroinflammation and contribute to the pathogenesis of various neurological disorders (Nayak et al., 2014; Salter and Stevens, 2017; Hickman et al., 2018). Understanding the complex functions of microglia in both health and disease is crucial for developing targeted therapeutic strategies for neurological disorders.

### 2.3 Activation states and phenotypes

**FIGURE 1:**
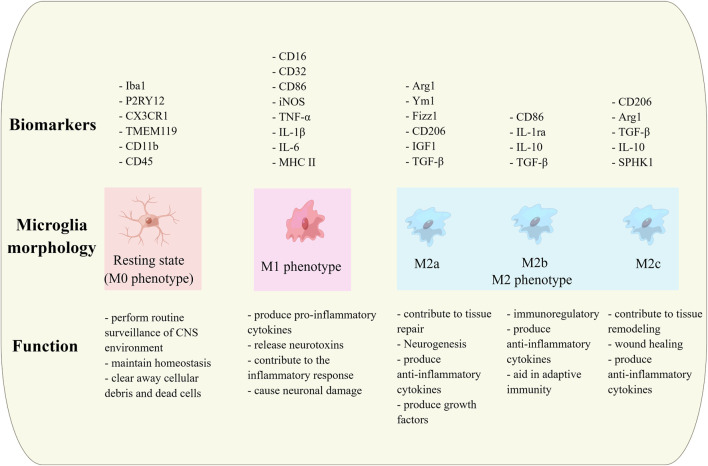
Biomarkers and diverse biological functions of microglia in different morphologies.

Microglia can be divided into 3 morphological forms, namely, branch-like, amoeboid and intermediate, according to their significant changes in morphology (Figure 1) (Savage et al., 2019). In turn, they can be classified into four types according to their functional state, namely, resting, intermediate, activated and aging states (Orihuela et al., 2016). In the resting state, microglia appear as small, branched cells with long protrusions. When the CNS is stimulated by inflammation or other injuries, microglia are activated and morphologically appear as amoeboid cells with larger cell bodies and shorter protrusions (Wolf et al., 2017; Orihuela et al., 2016).

Historically, microglial activation was simplified into two phenotypes: the “classic” pro-inflammatory M1 and “alternative” anti-inflammatory M2 states, representing extremes of microglial activation (Orihuela et al., 2016; Ransohoff, 2016a). However, it is now widely recognized that microglial activation is highly dynamic and context-dependent, with microglia adopting various activation states in response to different stimuli (Deczkowska et al., 2018). These activation states are characterized by specific gene expression profiles, functions, and secreted factors that can be either neuroprotective or neurotoxic (Bachiller et al., 2018; Spiteri et al., 2022). In general, resting-state microglia assist neuronal circuit formation and induce neuroactive synaptic plasticity by phagocytosing neuronal fragments (Nayak et al., 2014; Cornell et al., 2022). Microglia M1 are neurotoxic phenotype, as they release pro-inflammatory cytokines and various neurotoxins, such as IL-1β, IL-6, TNF-α, which may damage neurons and even induce neuronal death (Tang and Le, 2016). In M1 activation state, microglia can initiate a pro-inflammatory response, producing pro-inflammatory factors to eliminate foreign pathogens and promote an adaptive immune response (Tang and Le, 2016; Kwon and Koh, 2020). On the contrary, microglia M2 are neuroprotective phenotype, as they release neurotrophic factors promoting neuronal growth and tissue repair, such as IL-4, IL-10, BDNF, which protect neurons (Kwon and Koh, 2020). M2 forms can be further classified into M2a, M2b and M2c subtypes, where M2a could counteract neuroinflammation by secreting IL-10 and various neurotrophic factors to attenuate the pro-inflammatory response and stimulate tissue repair. M2b are associated with increased phagocytic and immunomodulatory activity and secrete large amounts of the anti-inflammatory factor IL-10 as well as the pro-inflammatory factors IL-1β, IL-6 and TNF-α. M2c secrete IL-10 and TGF-β and are associated with anti-inflammatory effects, tissue repair and neuronal debris removal (Orihuela et al., 2016; Pepe et al., 2014; Guo et al., 2022). During acute stimulation, the activation of microglia is primarily M2 phenotype, which is beneficial for neuronal survival. However, during chronic stimulation, the activation of microglia is primarily M1 phenotype, which promotes neuronal degeneration and death (Salter and Stevens, 2017; Mariani and Kielian, 2009; Orihuela et al., 2016).

In the context of viral encephalitis, microglial activation states are influenced by the type of virus, host-pathogen interactions, and host immune status (Waltl and Kalinke, 2022; Chen et al., 2019). The balance between the various activation states can determine the outcome of infection, with excessive pro-inflammatory responses leading to neuroinflammation and neuronal damage, whereas anti-inflammatory responses can promote tissue repair and maintain neuronal homeostasis (Waltl and Kalinke, 2022; Griffin et al., 1992; Smeyne et al., 2021).

## 3 Microglial involvement in viral encephalitis

### 3.1 Recognition of viral pathogens

Microglia are crucial in the recognition and defense against viral infections in the CNS. Microglial recognition of viral pathogens is a critical step in the CNS immune response against viral encephalitis (Borst et al., 2021). This recognition is mediated by pattern recognition receptors (PRRs) expressed on microglial cells, includding RIG-I-like receptors (RLRs), Toll-like receptors (TLRs), and NOD-like receptors (NLRs), which can sense viral pathogen-associated molecular patterns (PAMPs) (Fitzgerald and Kagan, 2020; Kawai and Akira, 2011; Li et al., 2021). Upon recognizing a viral PAMP, these PRRs initiate downstream signaling pathways leading to the activation of transcription factors like NF-κB and IRF3/7, in turn promoting the production of pro-inflammatory cytokines, chemokines and type I interferons (IFNs) (Takeuchi and Akira, 2010).

One important group of PRRs involved in viral pathogen recognition is TLRs. TLRs can detect viral nucleic acids or proteins in the CNS. For example, TLR3 can recognize double-stranded RNA, a common viral genetic material, while TLR7 and TLR8 can recognize single-stranded RNA (Hornung et al., 2008). The immune response against viral infection is facilitated by the activation of TLRs, which also stimulate downstream signaling pathways that encourage the release of pro-inflammatory cytokines and chemokines (Zhang et al., 2007). In addition to TLRs, microglia also express other PRRs such as RLRs and NLRs (Kigerl et al., 2014). RLRs, including RIG-I and MDA5, can recognize viral RNA molecules and activate antiviral immune responses (Kato et al., 2006; Shen et al., 2021). NLRs, on the other hand, can detect viral components in the cytosol and activate inflammatory signaling pathways (Tuladhar and Kanneganti, 2020). Upon activation of these PRRs, microglia initiate downstream signaling cascades that involve transcription factors, like NF-κB and IRF3/7 (Newton and Dixit, 2012). Activation of NF-κB and IRF3/7 can lead to the generation of pro-inflammatory cytokines, chemokines, and type I IFNs, which are important for mounting an effective antiviral immune response in the CNS (Takeuchi and Akira, 2010). Overall, microglia play a crucial role in recognizing viral pathogens in viral encephalitis through the expression of various PRRs. These PRRs are activated, which triggers the synthesis of immune mediators that aid in the CNS’s immunological response to viral infections.

### 3.2 Antiviral immune response

The antiviral immune response is a complex process involving various cells and molecules that work together to eliminate viral infections. Microglia are key players in mounting an antiviral immune response in the CNS by producing type I IFNs, which possess potent antiviral activity and can limit viral replication and spread (González-Navajas et al., 2012; Reinert et al., 2021). Furthermore, microglia can phagocytose viral particles and infected cells, presenting viral antigens to T cells and promoting the recruitment of peripheral immune cells to the site of infection (Kettenmann et al., 2011; Moseman et al., 2020). Additionally, microglia can secrete a variety of cytokines that modulate the adaptive immune response, such as IL-12, IL-23, and IL-27, ultimately shaping the host defense against viral infections (Chen et al., 2019; Jeong et al., 2022).

### 3.3 Microglia-mediated neuroinflammation

Whereas microglial activation is essential for mounting an effective antiviral response, exaggerated or chronic activation can lead to neuroinflammation and subsequent neuronal damage and dysfunction (Mariani and Kielian, 2009; Borst et al., 2021). Activated microglia release a wide range of pro-inflammatory molecules, includingTNF-α, IL-1β, IL-6, and nitric oxide (NO). These molecules can induce a cascade of inflammatory responses, recruiting other immune cells to the site of infection and promoting the production of additional inflammatory mediators. This sustained inflammatory environment can exacerbate the neuronal injury and contribute to the progression of viral encephalitis (Dheen et al., 2007; Chauhan et al., 2017; Kajimoto et al., 2021). Furthermore, the unrestrained activation of microglia and the overproduction of inflammatory mediators can disrupt the blood-brain barrier (BBB). The BBB is a specialized barrier that tightly regulates the exchange of molecules between blood circulation and brain tissue. Disruption of the BBB allows the infiltration of immune cells and inflammatory molecules into the brain, further amplifying the neuroinflammatory response and aggravating the disease process (Ronaldson and Davis, 2020; Ghoshal et al., 2007).

### 3.4 Contribution to blood-brain barrier disruption

BBB acts as a physical and metabolic barrier that separates the CNS from the peripheral circulation, protecting it from harmful substances and pathogens (Zhao et al., 2015; Daneman and Prat, 2015). Microglia-mediated neuroinflammation can contribute to BBB disruption during viral encephalitis through the overproduction of pro-inflammatory cytokines and chemokines, which can enhance the permeability of the BBB and facilitate the infiltration of peripheral immune cells into the CNS (Varatharaj and Galea, 2017). Furthermore, activated microglia can upregulate the expression of matrix metalloproteinases (MMPs), which can further degrade the extracellular matrix and tight junction proteins, leading to increased BBB permeability and CNS damage (Behl et al., 2021; Almutairi et al., 2016).

In viral encephalitis, disruption of the BBB plays a significant role in disease progression and severity. For example, in JEV infection, microglial activation has been shown to induce BBB disruption by upregulating MMP-3 and MMP-9 expression, thereby exacerbating CNS infiltration of peripheral immune cells and neuroinflammation (Ashraf et al., 2021). Similarly, BBB disruption has been implicated in the pathogenesis of other viral encephalitides, such as HSV-1 and WNV (Daniels et al., 2017; Wang et al., 2003).

Taken together, microglial activation plays a complex and multifaceted role in viral encephalitis, contributing to both the antiviral defense and the potentially detrimental inflammatory response. The balance between these opposing roles of microglia may determine the outcome of viral encephalitis, and further research is required to better understand the precise mechanisms underlying microglial involvement in these disease processes.

## 4 Dual role of microglia in viral encephalitis

**FIGURE 2:**
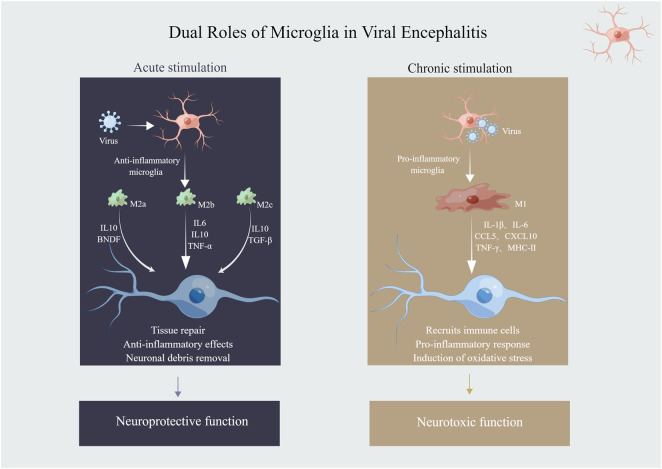
Dual roles of microglia in viral encephalitis.

Recent studies have fundamentally transformed our understanding of microglial biology in viral encephalitis contexts, moving beyond traditional models to more clinically relevant human-based systems.Human microglial responses to neurotropic viral infections exhibit profound species-specific differences that cannot be captured by traditional mouse models (Figure 2) (McMillan et al., 2023; Hasselmann et al., 2019; Hasselmann and Blurton-Jones, 2020). The comprehensive analysis demonstrated that immortalized human microglial cell lines (C20 and HMC3) are transcriptionally distinct from primary human microglia, induced pluripotent stem cell-derived microglia (iMGs), and monocyte-derived macrophages (Hasselmann and Blurton-Jones, 2020; Rajab et al., 2021; Seah et al., 2025). This finding challenges the widespread reliance on mouse models and cell lines for understanding human microglial pathophysiology. Critically, some work established that human stem cell-derived microglial models reveal fundamentally different virus-host interactions compared to traditional approaches. For instance, while HIV-1 directly infects microglia and establishes latency through a Bim-dependent mechanism (Castellano et al., 2017; Huang et al., 2011), other viruses like SARS-CoV-2 and WNV do not productively infect microglia but still elicit robust proinflammatory responses through pattern recognition receptors (Jeffries and Marriott, 2017; Cheeran et al., 2005; Song et al., 2021). Studies across multiple neurotropic viruses (HIV-1, ZIKV, JEV, WNV, HSV, and SARS-CoV-2) revealed virus-specific patterns of microglial activation that provide essential foundations for developing targeted therapeutic interventions (McMillan et al., 2023; Lum et al., 2017; Kumar et al., 2020; Jacob et al., 2020). Furthermore, xenotransplanted human microglia (xMGs) express homeostatic microglial markers such as EGR1, P2RY12, TMEM119, CX3CR1, and SALL1, which are not expressed or substantially downregulated *in vitro* (Hasselmann et al., 2019; Mancuso et al., 2024). The xenotransplantation studies in CSF1-humanized mice showed higher transcriptional similarities between xMGs and *ex vivo* human microglia compared to iMGs and cultured primary microglia, providing a more accurate model of human disease biology (Hasselmann and Blurton-Jones, 2020; Tang C. et al., 2025; Fattorelli et al., 2021).

Through comprehensive analysis of transcriptomic data, microglial responses to viral CNS infections represent a spectrum of activation states rather than discrete polarization categories (Waltl and Kalinke, 2022). The critical temporal dynamics whereby microglia provide essential protective functions during acute viral encephalitis through pathogen recognition via Toll-like receptors, RIG-I-like receptors, and cGAS-STING pathways (Takeuchi and Akira, 2010), antiviral defense initiation through type I interferon production (Katzilieris-Petras et al., 2022), and T cell activation via MHC I and II antigen presentation (Moseman et al., 2020; Goddery et al., 2021). However, the prolonged microglial activation leads to chronic neuroinflammation through excessive production of proinflammatory cytokines such as TNF-α, IL-1β, and IL-6, ultimately resulting in synaptic loss and long-term neurological sequelae (Moseman et al., 2020; Vasek et al., 2016). Critically, microglial-T cell crosstalk is essential for maintaining effective antiviral responses while preventing immunopathology (Herz et al., 2015). Microglia serve as local antigen-presenting cells that restimulate infiltrating virus-specific T cells, ensuring that only relevant antigen-specific T cells remain active within the CNS.

These studies converged on demonstrating pathogen-specific microglial responses that challenge previous generalizations. Primary human microglia infected with HSV-1 produce high levels of TNF-α, IL-1β, IP-10, and RANTES without viral amplification (Katzilieris-Petras et al., 2022), while ZIKV-infected microglia serve as viral reservoirs and transmit infectious particles to neurons (Lum et al., 2017). Similarly, microglia depletion during WNV infections leads to increased viral titers and mortality (Seitz et al., 2018), while during other viral infections, microglial activation may contribute more to pathology than protection. Together, these studies have shifted our understanding from viewing microglia as uniformly beneficial or detrimental toward recognizing their context-dependent, temporally dynamic, and virus-specific roles in viral encephalitis. This nuanced understanding provides the foundation for developing precision therapeutic approaches that can harness the protective functions of microglia while mitigating their pathological contributions, representing a significant advancement from the traditional binary view of microglial activation.

### 4.1 Neuroprotective functions

#### 4.1.1 Phagocytosis and clearance of viral particles

One of the key neuroprotective functions of microglia in the context of viral encephalitis is their ability to phagocytose and clear viral particles, thereby preventing viral spread and limiting neuronal damage. Microglia express a variety of PRRs that can detect PAMPs present in viral particles (Deczkowska et al., 2017). Upon recognition of PAMPs, microglia become activated, undergo morphological changes, and subsequently phagocytose the viral particles (Deczkowska et al., 2017; Kreutzberg, 1996).

Studies have demonstrated the importance of microglial phagocytosis in clearing viral particles and limiting CNS damage in animal models of viral encephalitis. For instance, during WNV infection, microglia play a significant role in clearing the virus from the brain, limiting viral replication, and ultimately reducing mortality (Stonedahl et al., 2020; Stonedahl et al., 2022). Similarly, in a murine model of HSV-1 encephalitis, microglial phagocytosis is essential for removing replicating viral particles from CNS neurons, thereby providing neuroprotective effects (Reinert et al., 2016; Marques et al., 2006). Recent advances in single-cell RNA sequencing have fundamentally challenged the traditional M1/M2 polarization, revealing instead that microglia exhibit a spectrum of activation states during viral infections (Schwabenland et al., 2021; Ransohoff, 2016b; Huang and Sabatini, 2020; Syage et al., 2020). Under homeostatic conditions, microglia consist of at least two different subpopulations, but during viral neuroinflammation, they demonstrate remarkable plasticity and heterogeneity (Borst et al., 2021; Hammond et al., 2019; Li et al., 2019). Despite these transcriptomic complexities, functional studies continue to identify M2-like microglial responses associated with neuroprotective outcomes. Treatment with anti-inflammatory agents like minocycline can reduce M1-like markers while promoting M2-like phenotypes that provide neuroprotective effects during viral encephalitis (Quick et al., 2017). During acute viral encephalitis, microglia function as the first line of defense against viral invasion within the CNS (Prinz and Priller, 2017). They provide essential pathogen recognition through pattern recognition receptors and initiate antiviral defense mechanisms via robust type I interferon production (Chhatbar et al., 2018; Detje et al., 2015; Drokhlyansky et al., 2017). Critically, microglia form physiological immune barriers that prevent viral dissemination to uninfected brain regions, effectively compartmentalizing infection and limiting tissue damage (Chhatbar et al., 2018). This barrier function is particularly evident in vesicular stomatitis virus infections, where microglia respond to locally produced type I interferons and create protective cellular networks (Detje et al., 2015). The temporal dynamics of microglial activation reveal context-dependent shifts between protective and potentially detrimental functions. However, sustained microglial activation can transition toward complement-mediated synaptic elimination and cognitive dysfunction during the recovery phase (Vasek et al., 2016). This temporal duality emphasizes that the same microglial populations can exhibit dramatically different functional outcomes depending on the phase of infection and duration of activation.

The importance of microglial phagocytic function in controlling the viral spread and contributing to the survival of the host suggests that modulation of microglial activity may represent a potential therapeutic strategy for the treatment of viral encephalitis. However, it should be noted that microglial phagocytosis may not be solely beneficial, as excessive phagocytosis of healthy neurons or synaptic elements may contribute to detrimental effects observed during CNS infection.

#### 4.1.2 Secretion of neurotrophic factors

Another neuroprotective function of microglia during viral encephalitis is the secretion of neurotrophic factors, which are capable of promoting neuronal survival, growth, and differentiation. During the early stages of CNS injury or infection, activated microglia have been shown to produce various neurotrophic factors such as NGF, BDNF, and GDNF (Rizzi et al., 2018; Prowse and Hayley, 2021; Zlotnik and Spittau, 2014).

For instance, BDNF secreted by microglia has been shown to enhance neuronal survival and neuroplasticity during viral infections such as HIV-1 encephalitis (Soontornniyomkij et al., 1998). In contrast, microglia often show a reduced capacity to produce neurotrophic factors during chronic inflammatory conditions, such as in neurodegenerative diseases and progressive viral infections. Dysregulated microglial function during these conditions may impair neuronal support and contribute to the progression of tissue damage (Calsolaro and Edison, 2016). Recent studies revealed unexpected neuroprotective effects of SARS-CoV-2 vaccination through enhanced microglial functions. Maternal SARS-CoV-2 vaccination during pregnancy enhances offspring hippocampal neurogenesis and working memory via IFN-γ–responsive microglia (Tang J. et al., 2025; Kumar et al., 2023). Inactivated SARS-CoV-2 vaccine administration during pregnancy led to transient enhancement of hippocampal neurogenesis in offspring at 1 month of age, mediated through microglial IFN-γR1 and CX3CR1 signaling pathways (Tang J. et al., 2025). The study revealed that microglia serve as critical mediators of vaccination-induced neuronal development through regulating microglial activation and chemotaxis. Conditional knockout experiments identified microglial IFNγR1 and CX3CR1 as key mediators, demonstrating that microglia activation following vaccination promotes neural precursor cell proliferation and neuronal differentiation through direct microglia-neuron interactions (Tang J. et al., 2025; Cserép et al., 2022). Importantly, the protective effects were temporally regulated and specific to the developmental period, with enhanced neurogenesis observed at 1 month but not at 2 months post-vaccination (Tang J. et al., 2025). This temporal specificity suggests that vaccination-induced microglial activation provides a beneficial developmental window for neuroplasticity without causing prolonged neuroinflammation.

The ability of microglia to secrete neurotrophic factors highlights the potential for stimulating their production as a therapeutic approach to promote neuronal survival and tissue repair during viral encephalitis. However, the precise mechanisms and signaling pathways involved in the regulation of neurotrophic factor secretion by microglia in the context of viral infections warrant further investigation.

#### 4.1.3 Regulation of neurogenesis

Additionally, microglia are essential in regulating neurogenesis, the process through which neural stem cells and progenitor cells turn into new neurons. During viral encephalitis, microglia may promote neurogenesis and contribute to tissue repair and functional recovery (Ekdahl et al., 2003; Ziv et al., 2006).

There are several ways through which microglia can regulate neurogenesis, such as by producing several regulatory molecules and signaling factors that influence neural stem cell proliferation, differentiation, and survival. These factors include IL-6, TGF-β, and IGF (Lu et al., 2011; Araki et al., 2021). Microglia can phagocytize cellular debris and apoptotic cells, thereby contributing to the clearance of detrimental factors and facilitating the regenerative process (Sierra et al., 2010). Altogether, the regulation of neurogenesis by activated microglia during viral encephalitis may represent a potential therapeutic target for facilitating CNS recovery. A better understanding of the molecular mechanisms and signaling pathways involved is required to develop suitable therapeutic strategies.

### 4.2 Neurotoxic functions

#### 4.2.1 Release of pro-inflammatory cytokines and chemokines

While microglia play a crucial role in the neuroprotective functions in viral encephalitis, they can also exert neurotoxic effects that contribute to neuronal damage and pathology progression. One of the primary neurotoxic mechanisms is the release of pro-inflammatory cytokines and chemokines during the activated state of microglia (Chen et al., 2019; González-Scarano and Baltuch, 1999; Rock et al., 2004).

Microglial activation resulting from viral infection or stimulation by viral components, such as viral proteins or nucleic acids, leads to the production and release of various pro-inflammatory cytokines, including TNF-α, IL-1β, and IL-6 (Dheen et al., 2007; Glass et al., 2010). These cytokines can contribute to neuronal dysfunction and death by exacerbating neuroinflammation, disrupting neuronal homeostasis, and enhancing oxidative stress (Allen and Barres, 2009; Lull and Block, 2010). Similarly, activated microglia can also release chemokines such as CCL2, CXCL10, and CX3CL1, that can mediate the recruitment of additional inflammatory cells, including peripheral immune cells, to the site of infection, thereby amplifying the inflammatory response and associated neurotoxicity (Errede et al., 2022; Kinuthia et al., 2020). In some cases, certain viruses can directly induce the production of these pro-inflammatory cytokines and chemokines by microglia, leading to augmented neurotoxic effects. For instance, infection with JEV in microglial cells has been shown to upregulate the expression of TNF-α, IL-6, and CCL2 (Ray and Ray, 2001; Verma et al., 2009). Similarly, HIV-1 proteins can stimulate the secretion of pro-inflammatory cytokines by activating microglia, thereby perpetuating neuroinflammatory damage (González-Scarano and Martín-García, 2005; Boerwinkle et al., 2021). Persistent SARS-CoV-2 spike protein components drive sustained neurodegeneration through pathological microglial activation. SARS-CoV-2 spike receptor-binding domain (RBD) drives sustained Parkinson’s disease progression via microglia-neuron crosstalk-mediated RTP801 upregulation (Wang et al., 2025a). RBD persistence in brain tissue accelerates dopaminergic neuron degeneration and α-synuclein aggregation through a pathogenic mtDNA-cGAS-STING-IFNβ/RTP801 feedback loop (Wang et al., 2025a; Wang et al., 2025b). RBD initially activates microglia, inducing neuronal RTP801 upregulation via IL-6 and IL-8 signaling (Wang et al., 2025a). Subsequently, RBD leads to microglial mitochondrial dysfunction, mtDNA release, and cGAS-STING pathway activation, establishing a self-perpetuating cycle of neuroinflammation and neurodegeneration. This pathogenic crosstalk between microglia and neurons amplifies PD pathology, with RTP801 serving as a critical mediator of RBD-induced neurodegeneration (Wang et al., 2025a). Targeting either RTP801 or microglial depletion significantly attenuated RBD-induced motor symptoms, cognitive impairment, and dopaminergic neuron loss (Wang et al., 2025a). Importantly, microglial depletion with PLX5622 prevented RBD-induced neurodegeneration, confirming the central role of pathological microglial activation in long-COVID neurological sequelae (Wang et al., 2025a; Solana-Balaguer et al., 2023).

Therefore, due to their capacity to release pro-inflammatory mediators, activated microglia can contribute to the pathogenesis of viral encephalitis by promoting neuroinflammation, neurotoxicity, and consequent neuronal damage.

#### 4.2.2 Induction of oxidative stress

Another neurotoxic function of microglia in viral encephalitis is the induction of oxidative stress. Activated microglia can produce reactive oxygen species (ROS) and reactive nitrogen species (RNS), which can damage cellular structures, including lipids, proteins, and nucleic acids, resulting in neuronal dysfunction and death (Lull and Block, 2010; Vezzani et al., 2016).

The activation of microglia following viral infection or exposure to viral components leads to increased production of ROS, such as superoxide anion and hydrogen peroxide (H2O2), through the activity of oxidative enzymes such as NADPH oxidase (NOX) (Xu et al., 2023; Ghosh and Basu, 2017; Sharma et al., 2021). Additionally, microglial activation also results in an increased production of RNS, such as nitric oxide (NO) and peroxynitrite (ONOO-), through the upregulation of inducible nitric oxide synthase (Katzilieris-Petras et al., 2022; Espinosa-Gongora et al., 2023; Klein et al., 2019). Various studies have reported the involvement of oxidative stress in the pathogenesis of viral encephalitis. For instance, in mouse models of West Nile virus infection, increased expression of iNOS and oxidative damage to neuronal cells have been observed (Wang et al., 2003; Kumar et al., 2010). Similarly, infection with HIV-1 leads to oxidative stress in neurons both *in vitro* and *in vivo*, partly mediated by increased iNOS expression and NO production in activated microglia (González-Scarano and Martín-García, 2005; Turchan et al., 2001).

Thus, by inducing oxidative stress, microglia can contribute to the neurotoxicity and pathogenesis of viral encephalitis, leading to neuronal dysfunction and cell death.

## 5 Microglial function in animal models of viral encephalitis

Recent advances in microglial depletion techniques have revolutionized our understanding of microglial function in viral encephalitis, revealing their unambiguously protective role across multiple viral infections (Hatton and Duncan, 2019). Through systematic analysis of mouse models employing colony-stimulating factor 1 receptor (CSF1R) inhibition, particularly using PLX5622 and related compounds, consistent findings have emerged that challenge traditional assumptions about neuroinflammation in viral encephalitis.

Microglial depletion studies across diverse neurotropic viruses demonstrate remarkable consistency in outcomes. Depletion of microglia via CSF1R inhibition invariably results in enhanced viral replication, increased mortality, and more severe neurological disease (Seitz et al., 2018; Hatton and Duncan, 2019). In flavivirus models specifically, microglial depletion led to 100% mortality in WNV-infected mice compared to 25% mortality in controls, accompanied by significant increases in viral RNA levels within the central nervous system (Seitz et al., 2018). Similar protective effects were observed in JEV infections, where PLX5622-treated mice showed significantly increased mortality compared to untreated controls (Seitz et al., 2018). The protective mechanisms appear multifaceted and extend beyond simple viral clearance. Microglia serve as essential antigen-presenting cells that restimulate virus-specific CD8^+^ T cells, ensuring effective immune surveillance within the CNS (Funk and Klein, 2019). Furthermore, microglial phagocytosis of infected neurons and viral debris represents a critical clearance mechanism, with P2Y12-mediated phagocytosis being particularly important in controlling viral spread (Fekete et al., 2018). In pseudorabies virus infections, microglia were observed to be recruited toward virus-infected neurons and actively engulf them through P2Y12 signaling (Fekete et al., 2018). The loss of this phagocytic function in microglial-depleted mice resulted in overt neurological disease and increased viral replication (Fekete et al., 2018). Paradoxically, microglial depletion often leads to enhanced rather than reduced neuroinflammation, suggesting that microglia exert regulatory control over excessive immune responses (Seitz et al., 2018; Hatton and Duncan, 2019). In WNV-infected, PLX5622-treated mice, several proinflammatory genes including CCL2, CCL7, CXCL9, and CXCL10 were actually upregulated compared to infected controls (Seitz et al., 2018), indicating that other CNS cells can produce these inflammatory mediators and that microglia may normally provide immunoregulatory functions.

Despite consistent protective outcomes, significant heterogeneity exists in microglial responses to different viral pathogens. Japanese encephalitis virus models demonstrate substantial variability, with mouse strain, age, virus strain, and inoculation route accounting for considerable experimental variation (Bharucha et al., 2022). Meta-regression analysis of 127 JEV studies encompassing 5,026 individual mice revealed that these factors explain approximately 61% of experimental variability, while 39% remains unexplained (Bharucha et al., 2022). Critically, the relationship between viral tropism and microglial function varies considerably. While WNV does not productively infect microglia *in vivo*, JEV readily infects these cells (Seitz et al., 2018; Chen et al., 2012). Interestingly, despite this fundamental difference in viral tropism, microglial protection remains essential in both scenarios (Seitz et al., 2018). In JEV infections, only PLX5622-treated mice showing overt clinical illness had detectable viral titers in the brain, whereas no virus was detected in control-fed animals at equivalent time points (Seitz et al., 2018), suggesting that microglia provide protection even when they themselves are viral targets. The temporal aspects of microglial protection reveal complex dynamics throughout disease progression. In WNV models, significantly increased viral loads were detected in microglia-depleted mice at multiple time points (6, 9, and 10 days post-infection), indicating sustained protective functions throughout the infection course (Seitz et al., 2018). The protective effect was most pronounced during early CNS invasion, with microglia apparently controlling initial viral seeding and subsequent replication (Seitz et al., 2018). Evidence from complement-microglia interactions suggests that while microglial activation may contribute to acute protection, dysregulated responses might contribute to longer-term neurological sequelae (Vasek et al., 2016; Wang et al., 2012). In attenuated WNV models, complement C3-mediated microglial phagocytosis of presynaptic neurons was associated with spatial orientation defects in recovered animals (Vasek et al., 2016), highlighting the potential trade-off between acute survival benefits and chronic neurological damage.

The mechanisms underlying microglial protection extend beyond direct antiviral effects. Microglia coordinate complex cellular networks involving T cell responses, with depletion studies revealing variable effects on different T cell populations across viral models (Hatton and Duncan, 2019). In mouse hepatitis virus infections, microglial depletion significantly reduced CD4^+^ T cell recruitment and IFNγ expression, while paradoxically reducing regulatory T cell populations (Wheeler et al., 2018). These findings suggest that microglia orchestrate balanced immune responses that optimize viral clearance while limiting immunopathology. Interferon signaling represents another critical pathway through which microglia mediate protection (Drokhlyansky et al., 2017; Detje et al., 2009). Type I interferon responses within the CNS create an intrinsic antiviral network, with microglia serving as both sensors and effectors of this system (Nayak et al., 2014). Astrocytes have been identified as major producers of IFNβ upon infection with neurotropic RNA viruses (Detje et al., 2009; Pfefferkorn et al., 2016), yet the coordination between microglial sensing and astroglial interferon production requires further investigation.

Current animal models face significant limitations in translating to human disease. Species-specific differences in immune responses, transcriptional regulation, and genetic factors between mice and humans represent fundamental barriers to translation (Hatton and Duncan, 2019). The incomplete reduction of microglia in some depletion models further complicates interpretation of protective mechanisms (Hatton and Duncan, 2019). Additionally, systematic analysis reveals concerning gaps in experimental rigor. Among JEV studies, no investigations reported sample size calculations, temperature control during experiments was rarely documented, and fewer than 50% included statements regarding randomization or blinding (Bharucha et al., 2022). These methodological limitations significantly impact the reliability and reproducibility of findings. The median quality score was only 10 out of 17 across established CAMARADES criteria (Bharucha et al., 2022), indicating substantial room for improvement in experimental design and reporting.

The disconnect between robust preclinical evidence and clinical application remains stark. Despite compelling animal model data demonstrating microglial protection, no current clinical trials investigate microglial-targeted therapies for viral encephalitis. This translational gap reflects broader challenges in neurovirology research, where the complexity of human microglial biology cannot be fully recapitulated in rodent systems (Davis, 2008). Off-target effects of microglial depletion compounds represent another translational concern. PLX5622 has been shown to affect macrophage populations in various tissues (Spangenberg et al., 2016), and variable effects on circulating monocytes have been reported across studies (Seitz et al., 2018; Hatton and Duncan, 2019). For flavivirus models where systemic viral replication precedes CNS invasion, systemic myeloid cell depletion might confound results by enhancing peripheral viral loads (Seitz et al., 2018). Future research priorities must include development of more specific microglial targeting approaches, advanced *in vivo* imaging techniques for real-time microglial tracking, and standardization of experimental protocols to reduce inter-laboratory variability. The integration of single-cell multi-omics approaches with spatially resolved transcriptomics offers particular promise for dissecting the regulatory networks governing microglial state transitions during viral infections (Li and Barres, 2018).

## 6 Therapeutic strategies targeting microglia in viral encephalitis

### 6.1 Direct inhibitors of microglial activation

Targeting microglia and modulating their activation can be a promising approach for developing therapeutic strategies in viral encephalitis. Different approaches that have been explored include inhibiting the harmful effects of microglial activation, while promoting their neuroprotective functions.

Minocycline, a second-generation tetracycline, has been shown to possess anti-inflammatory and neuroprotective effects, partly by inhibiting microglial activation (Wang et al., 2020; Ansari et al., 2022). In animal models of viral encephalitis, treatment with minocycline was demonstrated to reduce microglial activation, neuronal damage, and improve neurological outcomes (Richardson-Burns and Tyler, 2005; Kumar et al., 2009). Furthermore, the drug’s ability to penetrate the BBB makes it a potential candidate for clinical trials in patients with viral encephalitis. Cannabinoids, the bioactive compounds found in Cannabis sativa, have shown potential in modulating microglial activation and exerting anti-inflammatory effects (Mecha et al., 2013). In a murine model of virus encephalitis, treatment with a cannabinoid receptor agonist led to reduced microglial activation and decreased neuroinflammation, resulting in improved survival and reduced neurological deficits (Zhang et al., 2009; Solbrig et al., 2013). However, further studies are required to evaluate the safety and efficacy of cannabinoids in treating viral encephalitis. Inhibition of Colony-stimulating factor 1 receptor (CSF1R), a receptor involved in microglial survival and proliferation, has been shown to reduce microglial activation and neuroinflammation in various neurodegenerative diseases (Elmore et al., 2014; Olmos-Alonso et al., 2016). Recent research has revealed strain-specific effects of the CSF1R-microglia axis in the context of neurotropic viral infection, along with innate abnormalities in microglial antigen presentation and subsequent T cell crosstalk that increase vulnerability to neurotropic picornavirus infection. CSF1R antagonist also limits the local restimulation of antiviral CD8^+^ T cells (Funk and Klein, 2019; Sanchez et al., 2021). Although the therapeutic potential of CSF1R inhibitors in viral encephalitis has not been directly tested in clinical, it could provide an avenue to modulate microglial activation and reduce neuroinflammation.

While these approaches hold promise, much remains to be understood about the specific roles of microglia in different stages of viral encephalitis and how best to modulate them without compromising their defensive roles. Furthermore, concerns about the potential side effects of long-term microglial inhibition, such as increased susceptibility to infections, need to be carefully considered. With more research, a better understanding of microglial biology and function in viral encephalitis will undoubtedly open new avenues for therapeutic intervention. Challenges aside, the potential rewards for patients with viral encephalitis are too significant to ignore. In conclusion, modulating microglial activation is a promising approach in developing therapeutic strategies for viral encephalitis. Further research is needed to understand the balance between the beneficial and harmful roles of microglia and to identify novel targets and strategies for the treatment of viral encephalitis (Table 1).

**TABLE 1:** Therapeutic strategies targeting microglia in viral encephalitis.

Category	Therapeutic agent	Primary target/Mechanism	Site of action	Preclinical evidence	Clinical status	Ref
Direct Microglial Inhibitors						
	Minocycline	Microglial activation inhibition	Microglia	Reduced activation, neuronal damage, improved outcomes in viral encephalitis models	BBB-penetrant antibiotic; limited clinical trials in neurological conditions, none specifically for viral encephalitis	Quick et al. (2017), Prinz and Priller (2017), Chhatbar et al. (2018), Detje et al. (2015), Drokhlyansky et al. (2017), Rizzi et al. (2018), Prowse and Hayley (2021), Zlotnik and Spittau (2014), Soontornniyomkij et al. (1998), Calsolaro and Edison (2016), Tang et al. (2025b), Kumar et al. (2023), Cserép et al. (2022), Ekdahl et al. (2003), Ziv et al. (2006), Lu et al. (2011), Araki et al. (2021), González-Scarano and Baltuch (1999), Rock et al. (2004), Glass et al. (2010), Allen and Barres (2009), Lull and Block (2010), Errede et al. (2022), Kinuthia et al. (2020), Ray and Ray (2001), Verma et al. (2009), González-Scarano and Martín-García (2005), Boerwinkle et al. (2021), Wang et al. (2025a), Wang et al. (2025b), Solana-Balaguer et al. (2023), Vezzani et al. (2016), Xu et al. (2023), Ghosh and Basu (2017), Sharma et al. (2021), Espinosa-Gongora et al. (2023), Klein et al. (2019), Kumar et al. (2010), Turchan et al. (2001), Hatton and Duncan (2019), Funk and Klein (2019), Fekete et al. (2018)
	Cannabinoids (CBD, THC)	CB1/CB2 receptor modulation	Microglia, neurons	Reduced microglial activation, decreased neuroinflammation, improved survival in viral models	No clinical trials for viral encephalitis; regulatory challenges limit clinical investigation	Wang et al. (2012), Wheeler et al. (2018)
	CSF1R Inhibitors (PLX5622)	Colony-stimulating factor 1 receptor antagonism	Microglia	Microglial depletion, reduced neuroinflammation, strain-specific therapeutic effects	Preclinical research only; no clinical trials for viral encephalitis	Davis (2008), Spangenberg et al. (2016)
Cytokine/Mediator Blockade						
	Anti-IL-1β agents (Anakinra, Canakinumab)	Interleukin-1β signaling blockade	Microglia, immune cells	Reduced neuroinflammation, improved survival outcomes	FDA-approved biologics for autoimmune diseases; not tested in viral encephalitis	Ashraf et al. (2021)
	Anti-TNF-α agents (Infliximab, Etanercept)	Tumor necrosis factor-α neutralization	Microglia, immune cells	Decreased neuroinflammation, neuroprotection in viral models	FDA-approved biologics for rheumatoid arthritis/IBD; not tested in viral encephalitis	Li and Barres (2018), Wang et al. (2020)
	NF-κB Inhibitors (Bay 11–7,082, PDTC)	Nuclear factor κB pathway inhibition	Microglia, multiple cell types	Reduced pro-inflammatory gene expression, decreased microglial activation	Experimental compounds only; no clinical trials for any neurological indication	Richardson-Burns and Tyler (2005), Kumar et al. (2009), Mecha et al. (2013), Zhang et al. (2009), Solbrig et al. (2013)
	COX-2 Inhibitors (Celecoxib, Rofecoxib)	Cyclooxygenase-2 pathway inhibition	Microglia, inflammatory cells	Decreased prostaglandin production, anti-inflammatory effects	Most withdrawn from market due to cardiovascular risks; very limited current clinical use	Sköldenberg et al. (2006), Rubio-Perez and Morillas-Ruiz, (2012)
Neuroprotective Enhancers						
	PPAR-γ Agonists (Pioglitazone, Rosiglitazone)	Peroxisome proliferator-activated receptor γ activation	Microglia	M1→M2 phenotype shift, enhanced neuroprotection	FDA-approved for type 2 diabetes mellitus; investigational off-label use in neurological conditions	Oeckinghaus and Ghosh, (2009), Gu et al. (2012)
	Nrf2 Activators (Sulforaphane, Curcumin)	Nuclear factor erythroid 2-related factor 2 activation	Microglia, neurons	Enhanced antioxidant response, reduced ROS production	Available as dietary supplements only; no pharmaceutical formulations approved for neurological use	Tung et al. (2011)
	P2Y12 Agonists (2-MeSADP)	Purinergic receptor P2Y12 activation	Microglia	Enhanced microglial ramification, improved phagocytic function	Preclinical research stage only; no clinical development programs initiated	Chen et al. (2001), Ju et al. (2022), Chen et al. (2002), Bindu et al. (2020), Liu et al. (2021)
Immune Modulators						
	Corticosteroids (Dexamethasone, Prednisolone)	Glucocorticoid receptor activation	Microglia, immune cells	Broad anti-inflammatory effects, reduced microglial activation	Widely used clinically for various inflammatory conditions; limited evidence in viral encephalitis	Haynes et al. (2006), Suzuki et al. (2020)
	IVIG (Intravenous Immunoglobulin)	Multiple immune pathways modulation	Immune cells, microglia	Immunomodulation, reduced neuroinflammation	FDA-approved for various autoimmune conditions; case reports only in viral encephalitis	Burnstock, (2017), Imraish et al. (2023)
Combinatorial Approaches						
	Antiviral + Anti-inflammatory combinations	Multiple viral/immune targets	Virus, microglia, immune system	Enhanced therapeutic efficacy in JEV and other viral models	Individual components have regulatory approval for different indications; combinations not clinically tested	Omeragic et al. (2019), Ma, (2013)
	Glutamate Receptor Modulators (Memantine)	NMDA/AMPA receptor modulation	Neurons, microglia	Neuroprotection against excitotoxicity, reduced microglial activation	FDA-approved for Alzheimer’s disease; not tested in viral encephalitis	Cui et al. (2021)
	Neurotrophic Support (BDNF, GDNF, NGF)	Growth factor pathways	Neurons, microglia	Promoted neuronal survival, enhanced regeneration	Recombinant growth factors in early-phase clinical trials for other neurodegenerative diseases	Kraft et al. (2007), Schachtele et al. (2012)
	S1P Receptor Modulators (Fingolimod, Siponimod)	Sphingosine-1-phosphate signaling	Immune cells, CNS	Immunomodulation, reduced neuroinflammation, BBB protection	FDA-approved for multiple sclerosis; not investigated in viral encephalitis	Burnstock (2017)

### 6.2 Cytokine/mediator blockade

An alternative approach for developing therapeutic strategies in viral encephalitis is to inhibit the production or signaling of pro-inflammatory mediators that contribute to neuroinflammation and subsequent neuronal damage.

IL-1 and TNF-α are pro-inflammatory cytokines, produced primarily by activated microglia, that have been implicated in the pathogenesis of viral encephalitis (Sköldenberg et al., 2006; Rubio-Perez and Morillas-Ruiz, 2012). Inhibition of IL-1 and TNF-α has been reported to reduce neuroinflammation and improve outcomes in animal models of viral encephalitis (Ashraf et al., 2021; Niu et al., 2020; Zhang et al., 2017). Biological agents targeting these cytokines are in clinical use for other inflammatory conditions, thus providing a basis for their potential application in viral encephalitis. NF-κB signaling modulates the expression of genes encoding various pro-inflammatory mediators, including cytokines, chemokines, and adhesion molecules (Zhang et al., 2017; Oeckinghaus and Ghosh, 2009). Inhibition of NF-κB activation has shown promising results in reducing neuroinflammation in preclinical models of various neurological disorders, including viral encephalitis (Gu et al., 2012; Moniuszko-Malinowska et al., 2017; Wang et al., 2022). The development of specific inhibitors targeting key components of the NF-κB pathway may offer a novel therapeutic approach for viral encephalitis. Cyclooxygenase (COX) and lipoxygenase (LOX) enzymes mediate the production of pro-inflammatory lipid mediators, such as prostaglandins, thromboxanes, and leukotrienes, contributing to the neuroinflammation associated with viral encephalitis (Ghasemi et al., 2019; Tung et al., 2011; Chen et al., 2001). COX-2 inhibitors have been shown to decrease inflammation and improve outcomes in animal models of viral infection (Ju et al., 2022; Chen et al., 2002). However, given the cardiovascular side effects and organ damage associated with COX-2 inhibitors (Bindu et al., 2020), caution should be exercised in their application, and the development of more specific inhibitors targeting individual lipid mediators may hold therapeutic promise for viral encephalitis.

Hence, targeting pro-inflammatory mediators presents a potential therapeutic strategy for viral encephalitis. Further research is required to identify the most suitable candidates and determine their safety and efficacy in the context of viral encephalitis (Table 1).

### 6.3 Neuroprotective enhancers

Microglial activation during viral encephalitis can result in both detrimental and protective outcomes. Enhancing the neuroprotective functions of microglia, while limiting their neurotoxic effects, will inevitably be a promising approach for treating viral encephalitis.

Microglia display a spectrum of activation states that range between pro-inflammatory (M1 phenotype) and neuroprotective (M2 phenotype). Strategies that promote a shift from M1 to M2 phenotype have been shown to improve outcomes in various neurological disorders (Guo et al., 2022; Liu et al., 2021). In a model of HIV-1-associated brain inflammation, treatment with peroxisome proliferator-activated receptor gamma (PPAR-γ) agonists such as pioglitazone and rosiglitazone induced a neuroprotective M2 phenotype in microglia and reduced neuronal cell death (Omeragic et al., 2017; Omeragic et al., 2019). Nuclear factor erythroid 2-related factor 2 (Nrf2) is a transcription factor that regulates the expression of antioxidant and anti-inflammatory genes, which can improve the neuroprotective functions of microglia (Ma, 2013; Cui et al., 2021). Activation of the Nrf2 pathway has been shown to provide protection in various neuroinflammatory conditions, including neuroviral diseases such as ALS-like pathology in mice (Kraft et al., 2007). A study has revealed that sulforaphane treatment is effective in reducing neurotoxicity associated with HSV-stimulated microglia ROS production, as well as modulating neurotoxicity associated with experimental herpes encephalitis through sulforaphane treatment (Schachtele et al., 2012). The purinergic receptor P2Y12 has been identified as a potential target for modulating microglial function through the regulation of microglial ramification and motility (Haynes et al., 2006; Suzuki et al., 2020). Activation of P2Y12 receptors by selective agonists, such as ADP, reduces neuroinflammation and promotes phagocytosis of neuronal debris, conferring neuroprotection in various models of neurodegenerative diseases (Burnstock, 2017; Imraish et al., 2023; Liu et al., 2017). Further studies are required to investigate the role of P2Y12 receptor targeting in viral encephalitis.

To a certain degree, strategies that enhance the neuroprotective functions of microglia offer a promising therapeutic approach for viral encephalitis. Further research is required to validate these targets in the context of viral encephalitis and to identify additional strategies that may modulate microglial functions for neuroprotection (Table 1).

### 6.4 Combinatorial approaches

Combination therapies targeting multiple aspects of immune-inflammatory responses, including microglial modulation, viral replication, and neuronal support, could provide enhanced therapeutic benefits for viral encephalitis treatment in future clinical practice.

Antiviral treatments combined with anti-inflammatory drugs: The primary goal of treating viral encephalitis is to control and eliminate the underlying infection. Antiviral drugs such as acyclovir, ganciclovir, and foscarnet have proven effective against specific viral infections (Solomon et al., 2012; Wei et al., 2021). However, given the complex pathophysiology of viral encephalitis and the contribution of immune-mediated processes to tissue damage, combining antiviral agents with anti-inflammatory or immunomodulatory drugs could provide more comprehensive treatment strategies. For example, the combination of antiviral ribavirin with minocycline, an antibiotic with immunomodulatory properties, resulted in increased survival and reduced neuroinflammation during JEV infection (Topno and Khan, 2016; Joe et al., 2022).

Adjunctive therapies supporting neuronal survival: The prevention of neuronal loss is crucial for mitigating the neurological sequelae of viral encephalitis. Glutamate-mediated excitotoxicity is a common feature of many neuroinflammatory and neurodegenerative disorders, and targeting glutamate receptors with drugs such as memantine or riluzole could offer additional neuroprotection in viral encephalitis (Lipton, 2006). Furthermore, neurotrophic factors like BDNF and GDNF have shown potential in promoting neuronal survival and regeneration in various neurological conditions (Lima Giacobbo et al., 2019; Allen et al., 2013). Combining antiviral and anti-inflammatory agents with drugs supporting neuronal survival could represent a promising therapeutic approach in treating viral encephalitis.

Targeting endogenous neuroprotective signaling pathways: Besides directly targeting microglia, other endogenous neuroprotective pathways, such as Nrf2 (as mentioned above) and sphingosine-1-phosphate (S1P) signaling, could also be modulated to provide additional therapeutic benefits. S1P signaling has been shown to have a protective function in several neurological disorders by regulating immune cell trafficking, inflammatory gene expression, and cell survival (Czubowicz et al., 2019). Treatment with S1P receptor modulators, such as FTY720 (fingolimod), has demonstrated both immunomodulatory and neuroprotective effects in various preclinical models (Miron et al., 2010; McGinley and Cohen, 2021). Combination therapies incorporating S1P modulators and other targeted interventions might offer improved outcomes in viral encephalitis treatment.

Thus, combination therapies targeting multiple aspects of the immune response, viral replication, and neuronal support hold promise for the development of more effective therapeutic strategies for viral encephalitis. Further studies are needed to identify the optimal combinations of interventions and establish their safety and efficacy in treating various forms of viral encephalitis (Table 1).

## 7 Perspectives

Despite promising advancements in understanding the pathophysiology of viral encephalitis and the development of novel therapeutic strategies, several challenges and limitations remain, which impede the successful translation of these advancements into effective treatments for patients.

Microglia, the primary immune cells of the CNS, are essential players in the development and progression of viral encephalitis. Despite their importance, our understanding of microglial behavior during viral infection is still in its nascent stages due to several challenges and limitations. Primarily, the heterogeneity of microglia response to different viral pathogens adds to the complexity of the overarching mechanism. Microglia can adopt varied activation states, depending on the viral strain, each with distinct impacts on disease progression, which further complicates our understanding (Cherry et al., 2014). Besides, the lack of advanced *in vivo* imaging techniques for accurately visualizing microglia activation and interactions during disease progression is a notable limitation. This obstacle hinders real-time tracking of microglial behavior and disease progression within the CNS (Ransohoff and Perry, 2009). In addition, the successful treatment of viral encephalitis relies on a comprehensive understanding of host-pathogen interactions and immune responses (Jo, 2019; Jain et al., 2022). However, due to the complexity of these interactions and the diversity of causative pathogens, our knowledge remains limited, particularly regarding the underlying mechanisms that drive neurological damage and long-term sequelae. Further research is needed to interrogate host-pathogen interactions at the cellular and molecular levels to inform the development of targeted therapeutic strategies. Meanwhile, the development and optimization of therapeutic interventions for viral encephalitis rely on robust preclinical models that accurately recapitulate key aspects of the human disease. Although animal models have provided valuable insights into the pathophysiology of viral encephalitis, they often fail to recapitulate the full complexity of human disease due to differences in immune responses, transcriptional regulation, and genetic factors between species (Zschaler et al., 2014). Current clinical management of viral encephalitis relies primarily on antiviral agents and supportive care, with microglial-targeted therapies remaining largely theoretical despite promising preclinical evidence. Thus, the development of more accurate and representative preclinical models, including humanized mice and human organoid cultures, may facilitate the translation of promising therapeutic strategies into effective clinical treatments.

Single-cell RNA sequencing (scRNA-seq) and spatial transcriptomics are fundamentally transforming our understanding of microglial heterogeneity during viral encephalitis. These technologies have revealed unprecedented diversity in microglial transcriptional states that extend far beyond the traditional M1/M2 paradigm (Schwabenland et al., 2021; Huang and Sabatini, 2020), while spatial transcriptomics enables mapping of region-specific activation states within anatomical contexts (Han et al., 2024; Mallach et al., 2024). Future single-cell multi-omics approaches promise to reveal the regulatory networks governing microglial state transitions during viral infections. Additionally, a critical challenge is the significant disparity between microglial behavior *in vitro* and *in vivo* systems. Traditional microglial cultures fail to recapitulate the complex brain environment and crucial cell-cell interactions. While brain organoids and “brain-on-chip” models offer improved cellular interactions (Park et al., 2023; Zhang et al., 2023; Nandi et al., 2024), they still lack the full complexity of blood-brain barrier function and systemic inflammatory responses. Future research must develop hybrid approaches combining mechanistic insights from simplified systems with physiologically relevant *in vivo* validation.

Given the challenges, there is a need for strategic development and planning for future research directions. Improved *in vivo* imaging techniques to track the activation and interaction of microglia during disease progression would be pivotal in addressing the current limitations (Ransohoff and Perry, 2009). High-throughput single-cell analysis techniques can be employed to dissect the complex microglial heterogeneity and better understand the varied responses to different viral pathogens. Additionally, developing better animal models would be advantageous for simulating human viral encephalitis and studying the roles of microglia in the disease (Korin et al., 2017). Lastly, in-depth studies into the influence of other factors such as age, sex, and predisposing genetic factors on microglial activity during viral infection may open novel therapeutic avenues for viral encephalitis.
